# Social inequality, social networks, and health: a scoping review of research on health inequalities from a social network perspective

**DOI:** 10.1186/s12939-023-01876-9

**Published:** 2023-04-25

**Authors:** Sylvia Keim-Klärner, Philip Adebahr, Stefan Brandt, Markus Gamper, Andreas Klärner, André Knabe, Annett Kupfer, Britta Müller, Olaf Reis, Nico Vonneilich, Maxi A. Ganser, Charlotte de Bruyn, Holger von der Lippe

**Affiliations:** 1 Thünen Institute of Rural Studies, Bundesallee 64, Brunswick, Germany; 2 Institute of Sociology, University of Technology Chemnitz, Chemnitz, Germany; 3 Landesfrauenrat Mecklenburg-Vorpommern e.V., Rostock, Germany; 4 Institut für vergleichende Bildungsforschung und Sozialwissenschaften, University of Cologne, Cologne, Germany; 5 Rostocker Institut für Sozialforschung und gesellschaftliche Praxis e.V., Rostock, Germany; 6 Institute of Social Pedagogy, Social Work and Welfare Studies, Technische Universität Dresden, Dresden, Germany; 7 Institute of Medical Psychology and Medical Sociology, Rostock University Medical Center, Rostock, Germany; 8 Klinik für Psychiatrie, Neurologie, Psychosomatik und Psychotherapie im Kindes- und Jugendalter, Rostock University Medical Center, Rostock, Germany; 9 Institute of Medical Sociology, University Medical Center Hamburg-Eppendorf, Hamburg, Germany; 10 MSB Medical School Berlin, Berlin, Germany; 11 Alumna of MSB Medical School Berlin, Berlin, Germany

**Keywords:** Social network, Health, Inequality, Socioeconomic status, Social support, Scoping review, OECD

## Abstract

**Background:**

This review summarises the present state of research on health inequalities using a social network perspective, and it explores the available studies examining the interrelations of social inequality, social networks, and health.

**Methods:**

Using the strategy of a scoping review, as outlined by Arksey and O’Malley (Int J Sci Res Methodol 8:19–32, 2005), our team performed two searches across eight scientific, bibliographic databases including papers published until October 2021. Studies meeting pre-defined eligibility criteria were selected. The data were charted in a table, and then collated, summarised, and reported in this paper.

**Results:**

Our search provided a total of 15,237 initial hits. After deduplication (*n* = 6,168 studies) and the removal of hits that did not meet our baseline criteria (*n* = 8,767 studies), the remaining 302 full text articles were examined. This resulted in 25 articles being included in the present review, many of which focused on moderating or mediating network effects. Such effects were found in the majority of these studies, but not in all. Social networks were found to buffer the harsher effects of poverty on health, while specific network characteristics were shown to intensify or attenuate the health effects of social inequalities.

**Conclusions:**

Our review showed that the variables used for measuring health and social networks differed considerably across the selected studies. Thus, our attempt to establish a consensus of opinion across the included studies was not successful. Nevertheless, the usefulness of social network analysis in researching health inequalities and the employment of health-promoting interventions focusing on social relations was generally acknowledged in the studies. We close by suggesting ways to advance the research methodology, and argue for a greater orientation on theoretical models. We also call for the increased use of structural measures; the inclusion of measures on negative ties and interactions; and the use of more complex study designs, such as mixed-methods and longitudinal studies.

## Introduction

This scoping review departs from two meta-analytically substantiated insights into the sociological and psychological determinants of people’s health and health behaviour. First, there is broad evidence that *social inequality* engenders *health inequality*: the fewer economic and educational resources individuals possess, the less healthy they typically are, and the fewer – or less effective – health behaviours they exhibit [34, 50].

Second, the literature also indicates that *networks of personal relationships* are important *for individuals’ health and health behaviour* [3, 44]. Analyses of social networks take the structural and compositional features of people’s networks into account. These networks impact people’s health and health behaviour, with the effects ranging from helpful to harmful. For instance, networks that provide individuals with a high degree of social integration typically foster their well-being and promote social learning from their network members [44]. While these network relationships might lead people to adopt healthy behaviours, they might also push or entice people to engage in unhealthy risk behaviours. Thus, recent studies have examined all three aspects and their interrelationships,that is, they have addressed both social inequality and social networks in the context of research on health differences and inequalities.

In recent years, a large and growing number of empirical studies have examined the interrelationships between social networks and health. Although these findings are promising, research gaps have been pointed out [35, 38, 41]. What has so far been missing in this strand of research is a systematic exploration of how the social network perspective contributes to our understanding of the correlation between social and health inequalities.

From a theoretical point of view, the social network perspective has been identified as having the potential to improve our understanding of health inequalities [23]. This perspective may be particularly valuable when it focuses on the structures and the mechanisms that influence health outcomes, while also analysing how individual differences are multiplied by social networks, and how social inequalities are reproduced.

Therefore, this paper is guided by two leading questions:

- To what extent have existing studies examined social inequality, social networks, and health inequality using a single empirical approach?
- What have the findings of these studies revealed about the effects of the structural and the compositional characteristics of social networks on the association between social inequalities and health?

## The topic: social networks, health, and social inequalities

Today, the term “social network” is widely used, often in reference to online networks. However, our focus is on the research perspective of social network analysis that defines social networks broadly as “webs of social relationships that surround an individual and the characteristics of those ties” ([3], p. 145). Such relationships and their interconnections are not volatile,instead, they are arranged in “lasting structures” that are constantly produced and reproduced by interactions ([6], p. 6). It is this “overall configuration or pattern of relationships” ([44], p. 6) that is of interest in network research. This configuration may, for example, be captured by:

- *homogeneity* and *homophily measures* of one’s network members (indices of, respectively, the similarity of one’s network partners and their resemblance to oneself);
- measures of the *density* or *redundancy* of one’s network (indices that show to what extent a network is or is not loosely knit); or
- the presence and the number of bonding ties (the core network of close contacts) or bridging ties (which provide access to resources and information not available within the core network).

While these measures describe specific structural features, network indices form composite measures of different types of relations. An index that is often applied in this context is the Berkman-Syme Social Network Index (SNI), which focuses on the isolation/integration of a person, and collects information on the individual’s marital status, number and frequency of contacts with children, close relatives and close friends, church group membership, and membership in other community organisations. By means of such indices, subjects can be categorised into different network types, which are often characterised as having high to low levels of social integration [4].

An assumption that underlies many network approaches is that social networks “have emergent properties not explained by the constituent parts and not present in the parts” ([41], p. 407). Thus, network research combines an interest in the specific contacts individuals have in different areas of life (e.g., relatives, friends, colleagues,plus their characteristics) with a focus on their interactions and the functional aspects of these relationships (e.g., social support). Moreover, network research is interested in the larger structure that emerges based on these single ties. Drawing on this perspective, network research goes beyond other measures and concepts of social contacts and interpersonal influences by collecting information not only on the characteristics of the individuals in the network, but also on their relationships and their interconnections.

Social capital research from Bourdieu [8] to Coleman [13] or Putnam [36] has often referred to social networks in defining social capital. However, social capital studies have used structural network measures and network theory to varying degrees, from “building a network theory of social capital” [27], to focusing exclusively on the aggregate level, by, for example, measuring involvement in organisations and associations (yes/no) and general norms of trust [36]. These aggregate measures were often based on network theoretical thinking, with the aim of measuring network variables efficiently. For example, involvement in organisations can be used as an indicator of bridging ties without the need to collect complex network data.

Given the inconsistent and often metaphorical use of the term “social networks”, we have provided a definition of social networks that guides us in this review in Table 1. It specifies that social networks are characterised by relationship characteristics (focusing on different types of relations), by information on the function of relationships (e.g., support), and by structural information on the patterns the relationships form (e.g., density).

**Table 1:** Working definition of "social networks" for reviewing research on social networks and health inequalities

Social networks contain individual relationships of various types and with varying qualities, and can be characterised by:
1) relational information on relationships and their characteristics (e.g., on ties to relatives, friends, or neighbours; or on contact frequency or emotional closeness);
2) functional information (e.g., support, influence, conflict); and
3) structural information (e.g., size, density, homogeneity)

Source: own display

In health research, network studies examine the direct impacts of networks on people’s behaviour, as well as the diffusion of ideas, diseases, and behaviours within these structures [3, 44].

Unlike in health research, social network studies are not very common in inequality research [16], even though their concepts have much to offer in investigations of inequality [28]. Network characteristics such as homophily (“birds of a feather flock together”) or transitivity (“my friend’s friend is also my friend”) can lead to segregated networks of individuals who have either high or low resources, and can therefore not just reproduce social affiliations, but also foster social inequality and inhibit social mobility. People’s social status can affect their opportunities for engaging in beneficial social contacts. For example, an individual with higher social status has access to places (e.g., clubs, business meetings, societal events) where other people in higher social positions meet, and the person can use this access to social resources to advance his/her own career. In contrast, the homogenous network of an individual with lower social status cannot provide him/her with access to such resources [7]. These dynamics can be directly connected to an individual’s health and health behaviour, as people with higher social status are known to adopt healthier behaviours, and to form relationships with similar individuals, which may also have a positive impact on their health. This pattern can lead to the widening of health disparities [33]. However, the interrelations of social networks and social inequality are complex. Social networks can also help to reduce social inequality, by, for example, buffering individuals from the detrimental effects of financial deprivation [18]. The mechanisms involved in how social networks impact individual health or buffer or foster health inequalities are complex and manifold [24]. It is therefore far beyond the scope of this paper to go into all these possible mechanisms in more detail. In what follows, we focus on moderator and mediator effects of social networks on health inequalities.

**Fig. 1:**
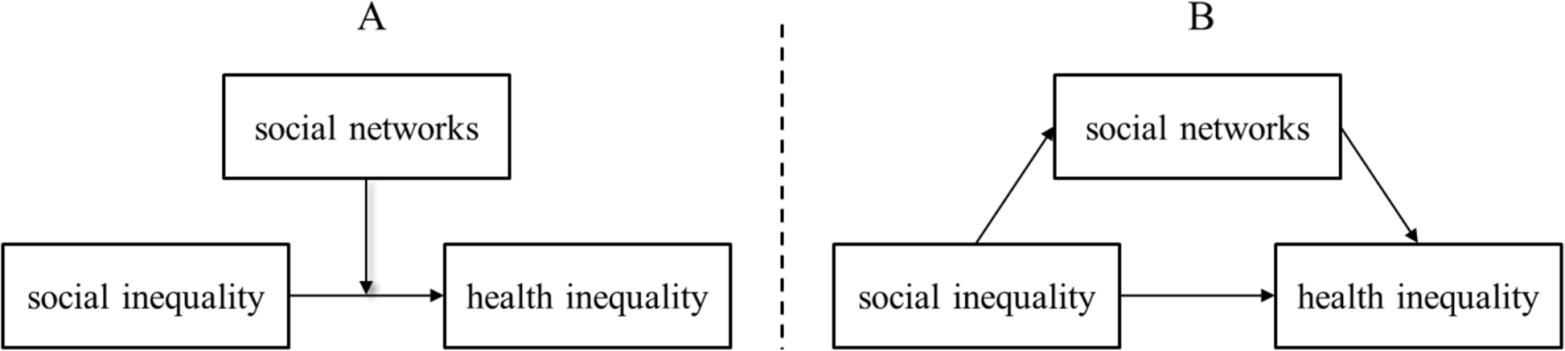
Schematic representation of moderator (**A**) and mediator (**B**) effects of social networks on health inequality. Source: own display

Figure 1 illustrates the most commonly identified types of interconnections between social networks and social and health inequalities. Part A of the figure displays a so-called moderator model. In this model, social networks do not have direct correlations with social or with health inequalities, but they can influence the correlations between these inequalities, by, for instance, having a buffer effect, as was mentioned above. Part B of the figure shows a so-called mediation model. Here, the baseline correlation between social and health inequalities is – fully or partially – altered (“mediated”) by the characteristics of a person’s social networks. In this model, the empirical question of whether the baseline correlation is attenuated (“statistical mediation”) or increased (“statistical suppression”) when social networks are included as a mediating variable remains open.

In the present scoping review, we examine to what extent existing studies have analysed the interconnections of social inequality, social networks, and health inequality using a single empirical approach. We also intend to summarise what the findings of these existing studies have revealed about the effects of the structural and the compositional characteristics of social networks on the association between social inequalities and health.

## Methods: reviewing studies using a social network perspective on health inequalities

The literature reviewing process was conducted by the members of a research network on social networks and health inequalities “Soziale Netzwerke und gesundheitliche Ungleichheiten (SoNegU)” funded by the German Research Foundation. The 25 members of this group discussed, scrutinised, and critically assessed the methodological approach and the proceedings of the review on several occasions, a total of 18 members were involved in the article screening. Although we did not strictly follow the PRESS strategy [30], we employed a third party review of our search strategy by two renowned experts in the field. Given the broad nature of our research questions and the heterogeneity of the studies in the field, we used the method of a scoping review. This method is especially applicable in contexts in which concepts are blurry or heterogeneously understood (as is the case for social networks, social support, social capital), and in which there are a variety of definitions, operationalisation approaches, and research designs that are difficult to compare systematically [1].

This scoping review strategy follows the method created and outlined by Arksey and O’Malley [1]. Their methodological framework describes five steps that must be taken to adequately perform a scoping review: 1. Identifying the research question; 2. Identifying relevant studies; 3. Selecting studies; 4. Charting the data; and 5. Collating, summarising, and reporting the results.

### Identifying relevant studies: data sources and search terms

The initial literature search was limited to peer-reviewed journal articles in English or German language, using the following international and German medical and social science data bases: Pubmed; PsycINFO and MEDLINE via Ovid; Solis (until 2017), SA (Sociological Abstracts, from 2017), SSA (Social Services Abstracts), ASSIA (Applied Social Sciences Index and Abstracts), and Scopus. The restriction to peer-reviewed articles was made for qualitative and time-efficiency reasons. Because the term "social networks" was often used metaphorically, we had to screen a large number of articles. A restriction to peer-reviewed articles ensured both a high-quality standard and a manageable number of hits. The search was conducted until October 2021. Articles were deemed eligible for inclusion only if they contained at least one search term from each of the following three topics: health, social inequality, and social network analysis. Table 2 displays the search terms that were applied to *titles*, *abstracts, keyword,* or *MeSH terms* of empirical studies*.* Additional information on how we translated these search terms into MeSH terms is provided in Appendix 1.

**Table 2:** Search terms of the initial retrieval step

Search Strategy: publications include at least one term from all three groups
**1) Health** *health, illness, disease, disorder, health status, health behavior, health behaviour, risk behavior, risk behaviour, coping, well-being, well-being, life satisfaction, mortality, morbidity, life expectancy, life expectancies, quality of life*
**2) Social inequality** *social status, socioeconomic status, socio-economic status, SES, social class, income, education, occupation, prestige, poverty, financial strain, wealth, low-income, deprivation, inequality, inequalities, disparity, disparities*
**3) Social network analysis** *social network, personal network, egocentered, egocentred, ego-centered, ego-centred, egocentric network, egonet, ego-net, whole network, family network, friendship network, support network, informal network, kinship network, network chart, network size, sociogram*

Source: own display

### Selecting studies: article screening and eligibility criteria

Eligibility criteria were consensually determined by the research group. Various preparatory steps (including a pilot trial) were taken to homogenise the evaluation of the literature in the research group (a two-stage pretest, discussion sessions for dissent, etc.). Through this process, a final eligibility list was generated.

Due to the large number of resulting findings, we needed to limit the scope of research to specific contexts. Given our interest in population health, studies focusing on health service use, on specific health issues (e.g., AIDS), or on specific groups (such as homeless individuals, or men who have sex with men) were excluded, as these works may require a separate review. A clear regional focus on OECD countries was also included in the criteria. Table 3 displays all of the study exclusion criteria.

**Table 3:** Exclusion criteria for articles not considered

Formal criteria:
duplicate, no journal article, not in English or German language, no empirical paper
Thematic criteria:
no focus on general human health (focus on health service use, specific health issues or specific groups), does not report vertical social inequality measures, does not treat personal networks (but instead treats neural networks or computer networks), no regional focus on OECD countries

Source: own display

**Fig. 2:**
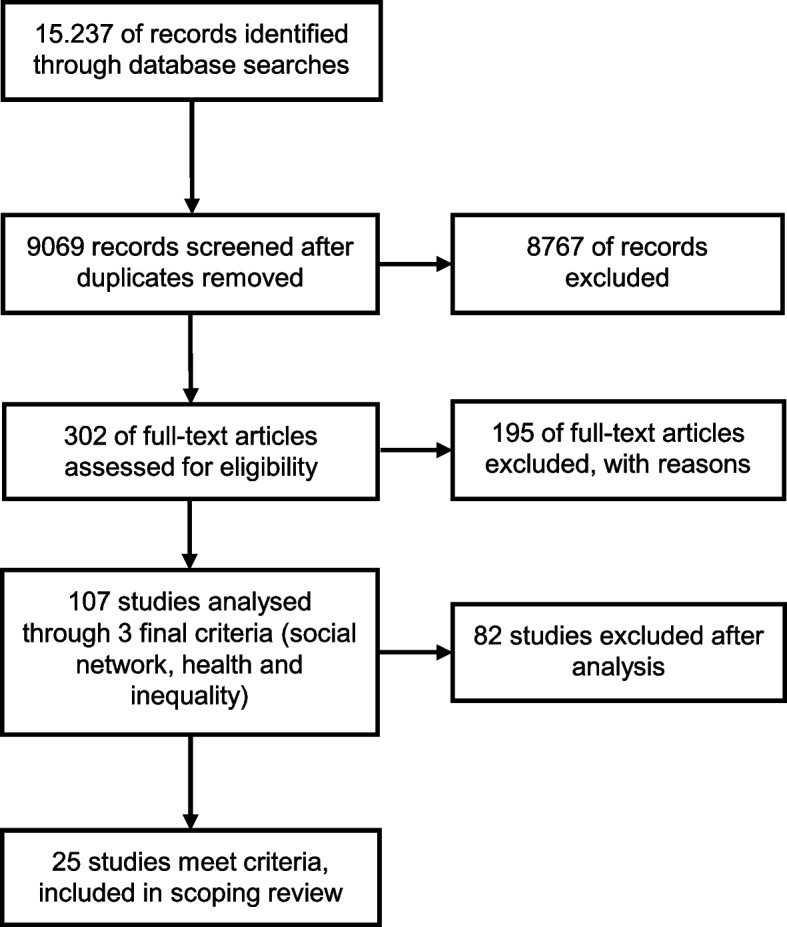
Flowchart of paper selection process (adapted from [1]). Source: own display

While we began the initial selection process by reviewing titles and abstracts, we then proceeded to review full texts (see Fig. 2). We recorded the selected texts, using a spreadsheet, indicating included or excluded texts as well as the reasons for exclusion. After reviewing full texts, it became obvious that some articles used the term “social network” in a rather metaphorical way, and often as a synonym for one-item measurements of social relations or support. We decided to exclude the studies that did not report on more advanced relational, functional, or structural indicators (see the aforementioned social network perspective). Additionally, the empirical connections the studies made between social inequality and social networks were evaluated. Papers that did not relate social inequalities to social networks, but instead treated them as independent controls, were excluded. Figure 2 displays the full sequence of the selection process.

### Data charting and final extraction

A resulting and refined 17-column spreadsheet indicated the relevant variables for extraction. This spreadsheet was pilot-tested with 20 papers, discussed in the working group and then provided to all involved in data charting along with examples and an explanatory text. The selected studies were evaluated with regard to characteristics such as research questions, study designs, methods and variables used, and central findings. Appendix 2 illustrates this procedure using the final study selection of this review.

## Results: characteristics and main findings of the eligible studies

### General characteristics of the final body of studies

The resulting 25 studies were conducted between 1999 and 2021. Of those, more than half were performed after 2010. Thus, a steady increase in the publication of relevant articles can be observed over the years, with only four of the studies being published before 2005. Of the selected studies, 15 were conducted in Europe, five were conducted in the US, two were conducted in Canada, two were conducted in Australia, and one was conducted in New Zealand.

Given our selection criteria, all 25 studies stem from academic journals, typically from health sector journals such as “Social Science and Medicine” (e.g., [9*, 45*]) and “Health & Place” (e.g., [2*, 15*, 46*]). None of the selected articles had been published in a journal on social network research. Although network journals publish various articles on health issues, these articles did not deal with social inequalities.

### Methodological characteristics of the final body of studies

#### Study design and methodology

Of the 25 included studies, 24 used quantitative data and performed one or more types of regression analyses. The studies were predominantly cross-sectional, and only seven papers used longitudinal data sets. One study used a mixed-methods design [2*] that began by collecting quantitative data through a postal questionnaire, followed by face-to-face interviews with participants from the questionnaire part. Cattell [9*] was the only purely qualitative study included in this review. Adopting a Grounded Theory approach, this study focused on in-depth face-to-face interviews in two impoverished London neighbourhoods, and developed a typology of social networks in relation to social and health inequalities.

#### Types of health measurements

The numbers and the types of variables collected in order to quantitatively depict health differentials varied between the studies (cf. also Appendix 2). A measure for self-rated, self-reported, subjective, or perceived health was most commonly used, appearing in 11 studies. Nine studies combined three or more health measures; e.g., on physical functioning, mental health, well-being, or vitality. Other studies focused on single measures of general interest, such as BMI (two studies) or resting heart rate (one study). Measures of health behaviour, such as smoking, alcohol consumption, or physical activity, were used as dependent variables in five studies.

#### Types of social inequality measurements

The included studies showed less variation in their approaches to measuring vertical social inequality. Most common were measures of education and income. Among the other measures used were wealth, perceived income adequacy, employment, occupation, social class, and economic living standard.

#### Types of social network measurements

The studies included in this review utilised a wide array of variables measuring social networks, including relational, functional, and structural measures (see Appendix 2). The relationship characteristics they collected were diverse, and most commonly referred to partnerships, family and friends, and contact frequency. In contrast, one functional characteristic, social support, was used in 15 of the studies. However, these studies varied considerably in the types of support they measured (e.g., emotional, instrumental), and in whether they assumed that the support was needed, perceived, received, or provided. Structural network measures were used in 15 studies, with the variable of network size being the most frequently used (11 studies). In contrast, the use of other structural network measures was rare, and those that were included were diverse. Network density was measured in three studies, and the homogeneity of networks was also measured in three studies. One study focused on homogeneity in terms of ethnicity and gender [20*], while another study focused on homogeneity of belonging to a small number of membership groups [9*], and a third study measured homogeneity of income, age, race, education, being employed, living in the local area, and being family members [26*]. Verhaeghe and colleagues [46*] studied the occupational composition of social networks. Homophily was measured in one study using the I-E Index, which indicated the similarity of alters with ego regarding smoking, age, and education [31*]. The structural constructs of bridging or bonding social relations were applied in three studies. In addition to network measures, 13 studies also used aggregate social capital measures such as trust, neighbourhood cohesion, or community activities.

Most studies covered two of the three named network characteristics: i.e., either relational and functional measures or relational and structural measures. Nine studies were more complex and used variables on multiple types of relationships, network functions, and structural information. For example, Nemeth and colleagues [31*] collected information on the persons the respondent spends the most time with and the persons whom the respondent asks for advice, including information on the characteristics of these persons (e.g., age, smoking status). They also used measures of perceived support (by partner, family, and friends), network size, and density. Four studies reduced complexity by using the Social Network Index (SNI) by Berkman and Syme [4] and the Social Integration Index (SII), a modified version of the SNI [5]. These indices captured different types of relations, and they were enriched with additional support or loneliness measures [25*, 29*, 47*, 48*, 49*].

### Main findings of included studies on the interconnections between social inequality, social networks, and health inequality

The interconnections between the crucial three constructs of interest were predominantly analysed using statistical moderator analyses (11 studies) and mediator analyses (nine studies), but multivariate regressions were also applied (six studies).^1^

#### Social networks as moderators of health inequalities (moderator analysis “type 1”)

Regarding the moderating effect of social networks on the correlation of social inequalities and health inequalities (here termed “type 1”; see Fig. 1A in the introductory section), the results of four out of five studies showed the relevance of different kinds of social network measures. Richards [37*] and Gele and Harsløff [20*] described moderator effects. Both focused on strong and weak network ties: on close ties, activities in organisations, and having a doctor as a friend [20*], and on friends, support, activities in organisations, and their frequency [37*]. Gele and Harsløff [20*] showed that in their sample, social networks were significant brokers of social resources. For example, they found that being linked to higher educated network partners was beneficial for the respondents’ health, particularly for those with low education. Richards [37*] observed that a high level of social integration acted as a buffer for the negative correlation of well-being and financial strains. Specifically, they found that financial problems impacted a person’s happiness less severely when the person had strong and supportive informal ties as well as extensive weak ties. In the studies by Baum et al. [2*] and Craveiro [15*], neither the relational, structural, nor functional network characteristics under study (e.g., contact frequency, support, network size) were shown to be relevant,but aggregate measures, such as perceived neighbourhood cohesion and safety, social participation, and general network satisfaction, were found to have a moderating effect on health inequalities. However, Craveiro [15*] observed these effects in central and southern Europe only. By contrast, Chappell and Funk [10*] found no similar moderator effects for network size, group membership, community activities, service use, or trust. Emotional support was only used as a control variable in this study.

#### SES as moderators of network impact on health (moderator analysis “type 2”)

In contrast to the model displayed in Fig. 1A, and somewhat surprisingly, six studies of the review sample also examined whether social inequality moderated the relationship between social networks and health (here termed “type 2” moderation). Four of these six studies found such moderating effects.

Schöllgen and colleagues [40*] reported that SES had a moderator effect on the relationship between social resources and health. Specifically, they found that network support was more beneficial for the subjective health of individuals with lower than with higher income levels. In a similar vein, Vonneilich et al. [47*] reported that the health risks associated with the lack of emotional and instrumental support were higher for subjects with lower than with higher SES. Focusing and bonding social capital, Kim et al. [22*] showed that the association between social capital and health was moderated by income, with higher bonding social capital being linked with better health for low-income households only. However, the measure of network density, in combination with the health measure BMI, was found to be associated with higher health risks for persons with lower levels of education [12*]. The authors explained this finding by noting that lower educated individuals tend to have less resourceful/supportive and more homophilic social networks. Unfortunately, measures of network homophily or support were not included in this study.

While Unger and colleagues [43*] and Weyers and colleagues [49*] did not find any “type 2” moderator effects in their data, they do no reject the relevance of social networks. The former authors assumed that it could be a mediator effect, and the latter authors stated that having a lower socio-economic position strengthens the influence of social networks on adverse health behaviour.

#### Social networks as mediators of health inequalities

Regarding the mediating effect of social networks on the correlation of social inequalities with health inequalities (see Fig. 1B), seven out of nine studies reported such findings. This mediating effect was found in cross-sectional studies as well as in longitudinal studies. The study by Li [26*] found that people of a higher social class were more likely to mobilise their social resources and networks for their own well-being than people of a lower social class. The authors noted, however, that these partial mediation effects of network size and diversity on health and well-being were numerically small compared to the effects of social class. In addition, Verhaeghe et al. [46*] found a prevalent class effect as well as a mediator effect of social networks, with lower levels of social support contributing to a reduction in self-rated health. In a longitudinal study, Klein and colleagues [25*] found that social networks mediated up to 35% of the social inequality effect on self-rated health. They showed that low SES was associated with lower levels of social integration and poorer social support, which in turn led to adverse effects on health. In their longitudinal study, Vonneilich et al. and colleagues [48*] also found a considerable mediating effect, stating that “social relationships substantially contribute to the explanation of SES differences in subjective health” ([48*]: 1/11).

In her qualitative study, Cattell [9*] described how being financially restricted or living in a poor area impeded social participation, which in turn led to a higher risk of social exclusion (smaller networks, less support) and poor health. She also showed that structural social network features (such as density and reciprocity) and functional characteristics (such as support) reduced the harsher effects of poverty on health.

While the mediation studies discussed so far dealt with some indicator of perceived or self-rated health, [29*] demonstrated that social isolation mediated the relationship between SES and resting heart rate by increasing the latter when subjects felt socially isolated and had smaller networks. Looking at different welfare regimes, Craveiro [14*] found that social networks mediated the effects of SES on health, but also that the mediating variables varied across different welfare regimes. However, social support and network satisfaction were found to be consistent mediators in all regions under study.

Two studies did not find any mediating effects. Chappell and Funk [10*] analysed the effects of network size, emotional support, group membership, and community activities on the perceived mental and physical health of individuals with different levels of education and income, while Sabbah and colleagues [39*] researched the mediating effects of support and network size on dental health. However, while the latter authors did not reject the idea that social networks could play a role in inequality in dental health in general, they critically evaluated their applied support measures. Among other measures, they used the subjects’ “need for emotional support”, instead of the more commonly used receipt of support.

#### Social networks in multivariate models of health inequalities

Another strand of studies refrained from formulating explicit moderator or mediator models. Instead, these six studies investigated social and health inequalities together with social networks in multivariate models without statistical interaction terms. All of these studies showed the relevance of social networks for health inequalities. Stephens and colleagues [42*] found that having a lower income was associated with having less social support, having a more restricted social network, and being less socially integrated, which in turn had detrimental health effects. Moreover, they found these social factors led to a higher risk of loneliness, which was strongly related to several adverse health effects. Similarly, Chavez et al. [11*] demonstrated that people who reported having trust and feelings of reciprocity in their social context had better self-reported health. Although Veenstra [45*] found that income and education were the strongest predictors for health, this author also showed that being socially integrated at work contributed to better self-reported health.

Two of these studies focused on health behaviours. Nemeth and colleagues [31*] showed that when strong neighbourhood cohesion was combined with the belief in the general acceptability of smoking in a deprived neighbourhood, and when social networks contained many smokers, it was more difficult for smokers to quit, as doing so would jeopardise their social acceptance. Kamphuis and colleagues [21*] showed that social networks played an important role in differences in sports participation, as people with lower SES, smaller networks, and less network cohesion had lower levels of sports participation.

The longitudinal study by Novak and colleagues [32*] introduced network characteristics from past study waves as “parental control in school” and “not being popular in school”. They showed that people who had low education as well as these social network characteristics during their school years were more likely to be obese in adulthood.

## Discussion and conclusion

Health inequalities have been investigated and discussed in research for many decades, and form an established research topic. Social network analysis, by contrast, is a novel research approach, but one that is gaining in popularity. Thus, the present scoping review was guided by two main questions: to what extent have existing studies examined social inequality, social networks, and health inequality using a joint empirical approach; and what have the findings of these studies revealed about the effects of the structural and the compositional characteristics of social networks on the association between social and health inequalities?

Despite the large number of initial hits in the literature research (9,064), after we reviewed the papers’ contents in more detail, we found a comparatively small number of publications (25) that fitted the aim of this review. A typical phenomenon that occurred during the selection process was that at first glance, many articles seemed suitable based on the term “social networks” in their abstract. At second glance, however, we found that the authors often used this term rather metaphorically for some unspecified kind of social relations or support. In these articles, which we excluded from this review, the authors neither made connections to the theoretical concepts or to methods of social network analysis; nor explored different types of relationships, analysed network functions, or measured structural network characteristics. Thus, despite the recent prominence of the term “social network”, there are only a few studies on health inequalities that can clearly be identified as social network analyses.

Among the studies considered for this review, the majority were published less than 10 years ago, and this set of studies exemplifies the growing popularity of network studies in research on health inequalities. From a theoretical and a statistical point of view, analyses of mediator, moderator, and multivariate models were the most common. Table 4 summarises the central results of this scoping review, which we will discuss in the following sections.

**Table 4:** Summary of central results of this review

(1) The theoretical bases of the studies differed considerably. Two types of moderator models could be distinguished from mediator and multivariate models
(2) The mediator and moderator models from Fig. 1 had the highest levels of plausibility and evidence, which provides support for the relevance of social network studies in research on health inequalities
(3) Social networks appeared to have the strongest effects on health inequalities when (i) they were observed in disadvantaged social contexts or societal strata; and (ii) they were studied in conjunction with health issues that were affected by social resources or capital
(4) The relevant allocation of social resources and capital to health inequalities was determined by the relational, functional, and structural characteristics of social networks

Source: own display

The empirical basis of statements 1 and 2 in Table 4 is provided by Table 5. When summarising the studies that applied statistical mediation or “type 1 moderation” models, we observed that 11 out of 14 empirical studies successfully located social networks *conceptually between* social and health inequalities. From our perspective, this approach also appears to be particularly valid, because networks temporally arise more frequently from a person's social class than vice versa. The models we termed “moderation type 2” models appeared to be less plausible to us for that reason. The 11 studies mentioned above showed that social networks are a relevant explanatory tool for improving our understanding of the complex relationships between socially unequal industrialised societies and health outcomes.

**Table 5:** Overview of the statistical and conceptual findings

Main statistical model	Number of studies that confirm expected effects	Number of studies that do not find effects	Total
Moderation type 1	4	1*	5
Moderation type 2	4	2	6
Mediation	7	2*	9
Multivariate analysis	6	-	6
Total	21	5	26

Chappell and Funk [10*] conducted both a mediation and a moderator analysis within one study (indicated by *). Source: own display

Another finding that we deem particularly interesting – including for future research – is that social networks appeared to be particularly relevant for attenuating the detrimental effects on health of deprived social contexts or statuses (statements 3 and 4 in Table 4). When the structure, function, or composition of social networks provide health-related resources to people, the health of deprived individuals typically benefits. However, we also found evidence of the often overlooked “dark side” of networks: studies that focused their research on behavioural variables to describe health (e.g., BMI, smoking, and alcohol consumption) often found a negative correlation between these behaviours and social inequality due to the *power and influence of social networks*. For instance, the study by Child et al. [12*] demonstrated that social networks could have reinforcing effects on the behaviour of people with both low and high incomes, resulting in higher predicted BMI for people in low-income communities and lower BMI for people in high-income communities. These results were echoed by Nemeth et al. [31*] in their study of smoking cessation and by Weyers et al. [49*] in their study of social relations’ overall impact on behaviour. In other words, depending on the variables used to describe health and networks, the intervening effects of networks on health changed, but the value of social network analysis within this research framework did not.

## Methodological discussion and future research directions

If the reported level of interest in the role of social networks in health research continues as was previously described, within the next few decades, more studies will be conducted using social network analysis as a mediator and moderator between health and social inequalities. This projected growth in the literature will provide a broader set of precedents and more consistency in both the variables collected and the results produced in this research field. While the 25 studies included in this scoping review provide an illuminating introduction to this topic, they should ultimately represent only the beginning of research into what we hope will become a very useful explanatory tool for studying these complex relationships in the years to come. Many of the studies in this review started out by stating that the body of knowledge on the role of social networks in health inequalities is small (e.g., [15*, 25*, 47*]), indeed, it appears that the authors of these studies were often unaware of other research on this topic, as the studies seldom cite each other in the text or in the references. This could reflect the strength of disciplinary boundaries; the centredness of the authors on specific health topics, populations, or specific countries; or the authors’ reading habits, which led them to focus on a small number of journals in the large landscape of health-related journals. This review can help scholars overcome these boundaries by bringing together evidence from various research streams.

Looking more closely at the methodological approaches and the network measures used in the presented studies provides us with valuable insights for formulating future directions for researching the role of social networks in health inequalities.

A first critical issue is the large amount of heterogeneity that the selected studies displayed, and especially the diversity of their network measures. Even a comparatively simple network variable such as size was measured in many different ways (number of close ties, number of supportive ties, etc.), and the studies often combined very different relational, functional, structural, and even aggregate measures into one empirical approach. This heterogeneity currently impedes quantitative meta-analyses in terms of systematic reviews. In future research on social and health inequalities, all network analyses could benefit by orienting empirical measures on theoretical models, such as the notions of supportive, integrating, influencing (norming), or contagious network ties. The current literature is still far from having reached a consensus on these issues. For instance, studies grounded in the theoretical considerations of social capital did not automatically enter this review because their network-related variables were typically too broad and too aggregate. While many of the included studies found that social networks could partially explain the association between socio-economic position and health, the researchers often did not observe the expected interrelations of social networks, social inequality, and health. These authors suggested [10*, 39*], or even insisted, that better measures should be used to clarify these interrelations [11*]. Apart from orienting measures on theoretical models in general, using structural network indicators may be one step in this direction, as the studies using multiple network variables indicated. The best examples of this approach were provided in the studies by Li [26*], who found mediating effects of network size and diversity, by [12*], who found a moderating effect of network density, and by [31*], who showed the relevance of homophily for smoking behaviour. One way of dealing with the complexities of collecting and analysing network measures is the development of tools such as the Social Network Index (SNI), which was created and developed by Berkman and Syme [4], or its successor, the Social Integration Index (SII) [5]. These tools were included in four of the studies presented here. They provide important precedents that constitute essential tools for increasing the consistency of studies in this field. In the future, the use of more standardised research tools would enhance the reliability and consistency of social network analysis within this research field. The benefits of using these tools would be compounded by the establishment of a set of baseline variables collected through precedent and testing.

Second, as social network research builds on a rich tradition of quantitative studies, it is not surprising that quantitative social network analysis emerges so strongly in the articles we reviewed. However, the single qualitative study included in the present study shows the valuable contributions qualitative methods can make in identifying the pathways through which social networks affect health, and the role of social inequality in this association. An especially interesting approach for future network research on social and health inequalities could be to conduct mixed-methods studies, which have received increased attention in recent years [17, 19]. Only one mixed-methods study entered this review, and the qualitative aspect of this study did not, unfortunately, focus on social networks.

Third, the studies we reviewed were mostly cross-sectional, and several of the authors explicitly asked for the use of more longitudinal data (e.g., [10*, 12*, 22*]). Fourth, another limitation of these studies that became apparent is the focus on the social gradient and the decision not to include certain socially excluded groups such as the homeless. Future studies could examine whether exploring the social networks of such groups would lead to similar conclusions.

Lastly, this research, which demonstrated how networks can buffer the detrimental health effects of having lower socio-economic status, or how networks pertain to the reproduction of health inequalities, can inform health policies in general, and health interventions more specifically. Many of the authors suggested focusing on social networks and on interventions aimed at helping socially disadvantaged individuals increase their contact with others. However, other researchers insisted that if we want to reduce health inequalities, we need to start by reducing social inequalities, given the evidence that accumulating ties that have detrimental effects on health (e.g., getting acquainted with other smokers) will not reduce health inequalities. Thus, when examining the effects of social networks on health inequalities, the role of negative ties and the negative effects of social relations on health inequalities should be considered more often.

## Data Availability

The datasets generated and analysed during the current study are available from the corresponding author on reasonable request.

## Appendix 1

**Table:** **Table 6** Translating search terms into mesh terms

*Search terms*	*MeSH terms*
***1. Topic: HEALTH***	
health, well-being	health
life satisfaction	no MeSH term
illness	disease attributes
disease, disorder	disease
health status	health status
health behavior	health behavior
risk behavior	risk-taking
coping	adaptation, psychological
mortality	mortality
morbidity	morbidity
life expectancy	life expectancy
quality of life	quality of life
**2. Topic: SOCIAL AND HEALTH INEQUALITY**	
social status, socioeconomic status, SES, social class, income, education, occupation, poverty, low-income, deprivation	socioeconomic factors
prestige	social class
wealth	- no MeSH term
financial strain	- no MeSH term
inequalities	socioeconomic factors, healthcare disparities
disparities	healthcare disparities, health status disparities
**3. Topic: SOCIAL NETWORK ANALYSIS**	
social network, support network, informal network	social support
personal network, egocentered, egonet, friendship network, egocentric network, whole network, network chart, sociogram	- no MeSH term
*family network, kinship network*	*family*

Comment: For our interest in Social Network Analysis (SNA), the MeSH terms *social support* and *family* are not sufficiently specific, therefore we did not use MeSH terms for the third topic SNA and rather performed our search with the search terms indicated above within titles and abstracts

## Appendix 2

**Table:** **Table 7** Data charting sheet: the 25 selected studies: study design and measurements

	**First author**	**Year**	**Study design**	**Health measures**	**Inequality measures**	**Network measures** **1) relational, 2) functional, 3) structural, 4) aggregate**	**Type of analysis**	**Main results**
1	Baum	2009	cross-sectional mixed methods	3: smoking, exercise, self-reported health	2: education, income	1) contact frequency 2) support 4)associational membership, reciprocity and 3 more	moderator analysis (type 1)	**moderating effect** of aggregate measures (neighbourhood cohesion, neighbourhood safety)
2	Cattell	2001	cross-sectional, qualitative	qualitative accounts on health and wellbeing	qualitative accounts on poverty	1) tie characteristics, contact frequency/duration 2) support given/ received 3) size, subgroups, density, homo/heterogeneity	qualitative mediator analysis	**mediating effect** (size, density, homogeneity, reciprocity, support)
3	Chappell	2010	cross-sectional, quantitative	3: perceived and mental health, physical function	2: education, income	3) size 4) trust, group membership, community activities, service use	moderator (type 1), mediator analysis	no moderating effect no mediating effect
4	Chavez	2004	cross-sectional, quantitative	1: self-reported health	3: housing, education, employment status	1) family/friends in neighbourhood (bonding ties) 2) support 4) trust, local participation/ membership and 8 more	multivariate analysis	trust is of relevance for health in disadvantaged neighbourhoods
5	Child	2018	cross-sectional, quantitative	1: BMI	2: education, income	1) up to three close ties, their educational level and residence 3) density	moderator analysis (type 2)	**moderating effect** of education on the association between network (density) and health
6	Craveiro	2016	cross-sectional, quantitative	3: chronic disease, perceived health, ADL limitation	4: education, income, wealth, perceived income adequacy	1) close ties, daily contact 2) support 3) size 4) social participation, network satisfaction	mediator analysis	**mediating effect** (participation, daily contact, support); mediator effects of size only in eastern countries
7	Craveiro	2017	cross-sectional, quantitative	3: chronic disease, perceived health, ADL limitation	4: education, income, wealth, perceived income adequacy	1) partner, children, daily contacts, closeness 2) support 3) size 4) participation in activities, network satisfaction	moderator analysis (type 1)	**moderating effect** in central and southern Europe for aggregate measures (mainly: social participation, network satisfaction), not in northern Europe
8	Gele	2010	cross-sectional, quantitative	1: self-rated health	3: occupation, income group and education	1)strong and weak ties, contact frequency w/ family and friends, nurses/medical doctors as friends 3)size, ethnic/gender diversity, bonding/bridging ties 4)activities in organisations	moderator analysis (type 1)	**moderating effect** (strong ties, activities in organisations, one friend is a doctor)
9	Kamphuis	2008	cross-sectional, quantitative	3: sports frequency, duration, intensity	2: education, income	3) neighbourhood network (small, medium, large) 4) social cohesion, opinion of neighbourhood, disorganisation, frequency of social gathering	mediator analysis	**mediating effect** (network size, neighbourhood attractiveness and neighbourhood safety, social cohesion)
10	Kim	2021	cross-sectional, quantitative	2: Self-rated health, physical activities	2: education, income	3) bonding and bridging ties (Personal Social Capital Scale)	moderator analysis (type 2)	**moderating effect** of income on the association of network (bridging and bonding ties) and health
11	Klein	2012	longitudinal, quantitative	1: self-rated health	3: income, education, profession	1 + 4) Social Integration Index: living with a partner, number of close ties, affiliation in associations 2) support	mediator analysis	**mediating effect** (SII and support)
12	Li	2016	longitudinal, quantitative	3: perceived health, happiness, satisfaction	3: degree and employment, parents’ class	3) size, diversity (income, age, race, education, employment, place of living, family member) 4) civic engagement, neighbourhood cohesion	mediator analysis	**mediating effect,** significant but small (size, diversity, neighbourhood cohesion, civic engagement)
13	McCrory	2014	cross-sectional, quantitative	1: resting heartrate,	1: household income	1 + 4) Social Network Index: marital status, number of children, contact frequency, close relatives/friends, church group/ organisational membership 4) loneliness	mediator analysis	**mediating effect** (SNI, loneliness)
14	Nemeth	2018	cross-sectional, quantitative	2: smoking, depression	3: education, income, employment status	1) partner, persons spending time with/asking for advice, their characteristics (e.g., smoking status) 2) perceived support, social influence scale, 3) size, density, E/I index (similarity of Ego and Alter in smoking, age and education) 4) participation, neighbourhood cohesion and 3 more	multivariate analysis	perception of social acceptability of smoking, homophily on smoking and neighbourhood cohesion are associated with current smoking in a disadvantaged population, cessation interventions need to acknowledge the social context
15	Novak	2006	longitudinal, quantitative	1: BMI	2: occupation (parents’ and own) and education	1) marital status, number of children, social network index, contact with parents; 2) social support index, parental support in adolescence 4) participation in associations; popularity in school	multivariate analysis	educational gradient in BMI at middle age is explained by support (men), popularity (women) in adolescence and nonparticipation in associations in young adulthood (men)
16	Richards	2015	longitudinal study, quantitative	2: long-term illness, subjective well being	2: financial situation, employment status	1) friends (strong ties) 2) support in 5 dimensions (strong ties) 4) activities in organisations, their frequency (weak ties)	moderator analysis (type 1)	**moderating effect** (weak ties, supportive strong ties)
17	Sabbah	2011	cross-sectional study, quantitative	2: damaged teeth, paradontose	2: education, income	1) close friends, marital status 2) support 3) size	mediator analysis	no mediating effect
18	Schöllgen	2011	cross-sectional study, quantitative	3: physical, functional, subjective health	2: education, income	2) emotional support, informational support 3) size	moderator analysis (type 2)	**moderating effect** (supportive network)
19	Stephens	2011	cross-sectional, qualitative	8: physical function, mental health, pain and more	2: economic living standard, education	1) living distance from relatives, contact frequency to family, neighbours, friends 2) support, 4) involvement in community, loneliness	multivariate analysis	social network and social support explain 33% of mental health and 15% of physical health, social gradients in health can be partially explained by social networks
20	Unger	1999	longitudinal, quantitative	1: physical functioning	2: education, income	1) marital status 2) emotional and instrumental support by spouse, children, friends and relatives 3) size	moderator analysis (type 2)	no moderating effect of income on the association of networks/support and functional decline
21	Veenstra:	2000	cross-sectional, quantitative	1: self reported health	2: income, education	1) contact frequency (family, workmates, neighbours…) 4) trust, religious attendance, civic participation	multivariate analysis	income and education were strongest predictors for health, social integration at work and attending religious services additionally contributed to better health
22	Verhaeghe	2012	cross-sectional, quantitative	1: self-rated health	3: education, occupation, social class	1) friends, family, acquaintances w/ different occupations 2) perceived support 3) volume of social capital, occupational composition	mediator analysis	positive associations between network social capital and health beyond the influence of social class. Support only partially mediated the association of network social capital and health
23	Vonneilich	2011	longitudinal, quantitative	2: self-rated health, depressive symptoms	2: income, education	1 + 4) Social Integration index: living with a partner, number of close ties, affiliation in associations 2) emotional/instrumental, perceived/received support	moderator analysis (type 2)	**moderating effect** of SES on the association between networks (SII, support) and health
24	Vonneilich	2012	longitudinal, quantitative	1: General subjective Health	3: income, education	1 + 4) Social Integration index: living with a partner, number of close ties, affiliation in associations ( 2) emotional/instrumental, perceived/received support	mediator analysis	**mediating effect** (SII, support)—> network interventions fostering relationships can help lower SES groups to enhance health and buffer health inequalities
25	Weyers	2010	cross-sectional, quantitative	3: smoking, nutrition, physical exercise	2 income, education	1) Social Integration index: living with a partner, number of close ties, affiliation in associations 2) emotional/instrumental, perceived/received support	moderator analysis (type 2)	no moderating effect; additive effect (SII, support)

## Footnotes

1 Numbers sum up to more than 25 or 100 percent, respectively, because one study presented more than one analytical approach.
